# Reproducibility with repeat CT in radiomics study for rectal cancer

**DOI:** 10.18632/oncotarget.12199

**Published:** 2016-09-22

**Authors:** Panpan Hu, Jiazhou Wang, Haoyu Zhong, Zhen Zhou, Lijun Shen, Weigang Hu, Zhen Zhang

**Affiliations:** ^1^ Department of Radiotherapy, Fudan University Shanghai Cancer Center, Shanghai, China; ^2^ Department of Oncology, Fudan University Shanghai Medical College, Shanghai, China

**Keywords:** radiomics, rectal cancer, reproducibility, textural features

## Abstract

**Purpose:**

To evaluate the reproducibility of radiomics features by repeating computed tomographic (CT) scans in rectal cancer. To choose stable radiomics features for rectal cancer.

**Results:**

Volume normalized features are much more reproducible than unnormalized features. The average value of all slices is the most reproducible feature type in rectal cancer. Different filters have little effect for the reproducibility of radiomics features. For the average type features, 496 out of 775 features showed high reproducibility (ICC ≥ 0.8), 225 out of 775 features showed medium reproducibility (0.8 > ICC ≥ 0.5) and 54 out of 775 features showed low reproducibility (ICC < 0.5).

**Methods:**

40 rectal cancer patients with stage II were enrolled in this study, each of whom underwent two CT scans within average 8.7 days. 775 radiomics features were defined in this study. For each features, five different values (value from the largest slice, maximum value, minimum value, average value of all slices and value from superposed intermediate matrix) were extracted. Meanwhile a LOG filter with different parameters was applied to these images to find stable filter value. Concordance correlation coefficients (CCC) and inter-class correlation coefficients (ICC) of two CT scans were calculated to assess the reproducibility, based on original features and volume normalized features.

**Conclusions:**

Features are recommended to be normalized to volume in radiomics analysis. The average type radiomics features are the most stable features in rectal cancer. Further analysis of these features of rectal cancer can be warranted for treatment monitoring and prognosis prediction.

## INTRODUCTION

As human oncology has a strong phenotypic difference from normal tissue, which may be visualized non-invasively by different imaging modalities, such as X-ray computed tomography (CT), positron emission tomography (PET), and magnetic resonance imaging (MRI). Among them, X-ray CT is the most widely applied in oncology, which can assess tissue density in high resolution and exhibit strong contrasts among different tissue types [1]. Nowadays, radiomics become a novel approach because it can utilize medical imaging to quantify the tumor phenotype non-invasively for further study, such as patients' survival, treatment monitoring and outcome prediction [2].

To get a reliable and reproducible result in radiomics study, it is essential to guarantee the repeatability of features extraction process. Recent publications have demonstrated that radiomics features be reproducibly measured from CT images for patients with non-small cell lung cancer [3]. And for nonsmall cell lung cancer (NSCLC) patients, quantitative image features extracted from computed tomography (CT) images can be used to improve tumor diagnosis, staging, and response assessment [4]. However, no studies have yet examined the stability and reproducibility of CT images textural features for rectal cancer. In addition, no researches have been conducted to analyze which kind of extraction process is the best, including max slice, max value, min value, average value or matrix sum.

Specifically, max slice means textural features from the slice with the largest GTV area of target images; max value means textural features from all slices of target images and the maximum value was selected; min value means textural features from all slices of target images and the minimum value was selected; average value means textural features from all slices of target images and average all values; and matrix sum means all slices of target images translated into GLCM [5] (gray level co-occurrence matrix) matrices and GLRLM [6] (gray level run-length matrix) matrices and superpose all matrices, then extract textural features from the superposed matrices.

Furthermore, in these researches, patients were found who had two sets of CBCT images obtained within 15 minutes, such a short time may not analyze the reproducibility of radiomics.

In this study, we analyzed the stability and reproducibility of radiomics features derived from manually segmented rectal tumors in forty patients who underwent two baseline clinical CT scan within average 8.7 days (5 days to 17 days), without any treatment before. Also, we evaluated the five values for each features to find out the most stable value.

## RESULTS

Table 1 presents the comparisons of geometry features (volume, area and volume/area) between two scans. It indicates that tumor volume increased 6% with an average 8.7 days' interval.

**Table 1:** Geometry features comparisons between two scans

	Volume (mm^3^)	Area (mm^2^)	V/A (mm)
Scan 1	45370.8 ± 35271.7	10921.1 ± 5246.5	3.9 ± 1.2
Scan 2	48002.5 ± 28625.2	10762.3 ± 4712.5	4.2 ± 0.9

**Figure 1:**
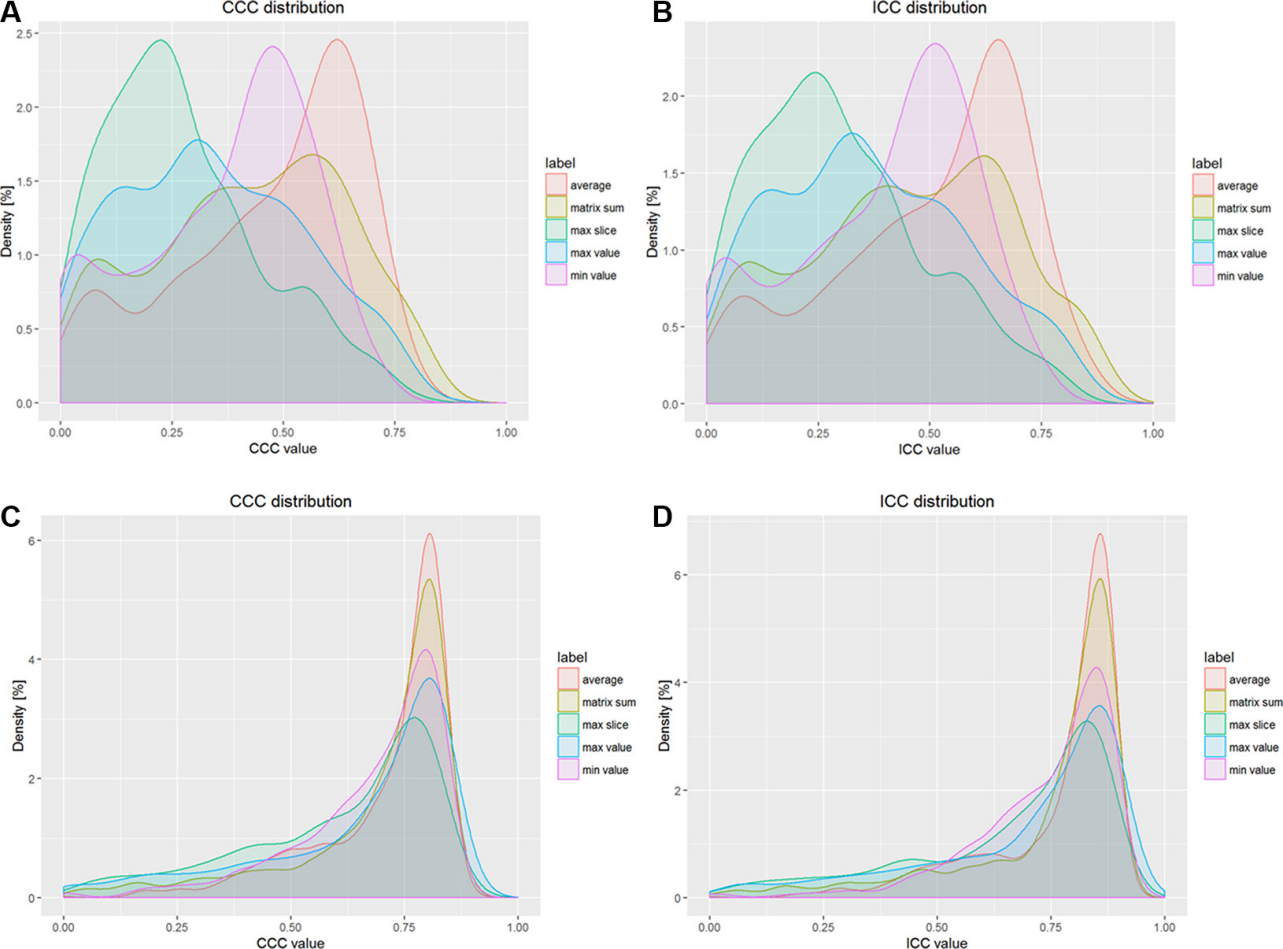
Results of CCC and ICC distributions for different types of features (**A**) Probability density of CCC distribution for unnormalized features, (**B**) Probability density of ICC distribution for unnormalized features, (**C**) Probability density of CCC distribution for normalized features, (**D**) Probability density of ICC distribution for normalized features.

**Figure 2:**
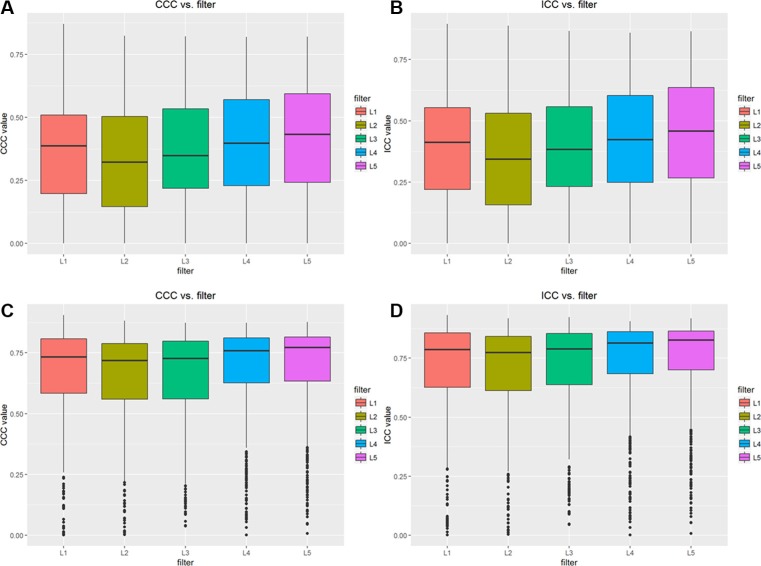
Results of CCC and ICC for different type of features with different filters (**A**) CCC values for unnormalized features, (**B**) ICC values for unnormalized features, (**C**) CCC values for normalized features, (**D**) ICC values for normalized features.

The results of CCC and ICC for different numerical types of unnormalized features and normalized features are listed in the Figure 1 (A, B) and (C, D) respectively:

In Figures 1 (A) and (B), the peak values of probability density for different types of original features are less than 2.5%, but in Figures 1 (C) and (D), the peak values of volume normalized features are more than 6%. For Figures 2 (A) and (B), the mean CCC values and mean ICC values for different types of original features are less than 0.40 and 0.45, but for in Figures 1 (C) and (D), the values are about 0.80 and 0.85, respectively. In Figures 1 (C) and (D), the peak density value and the mean value for average type are larger than other four types' features, so the average type volume normalized features are more reproducible than others.

The mean values of CCC and ICC for different filters of original features are less than 0.45 showed Figures 2 (A) and (B), however the mean values of CCC and ICC for different filters of volume normalized features are 0.75 and 0.80, respectively, showed Figure 2 (C) and (D). Furthermore, no statistically significant difference was observed in CCC and ICC values for different filters, showed in Figure 2 (C) and (D).

**Figure 3:**
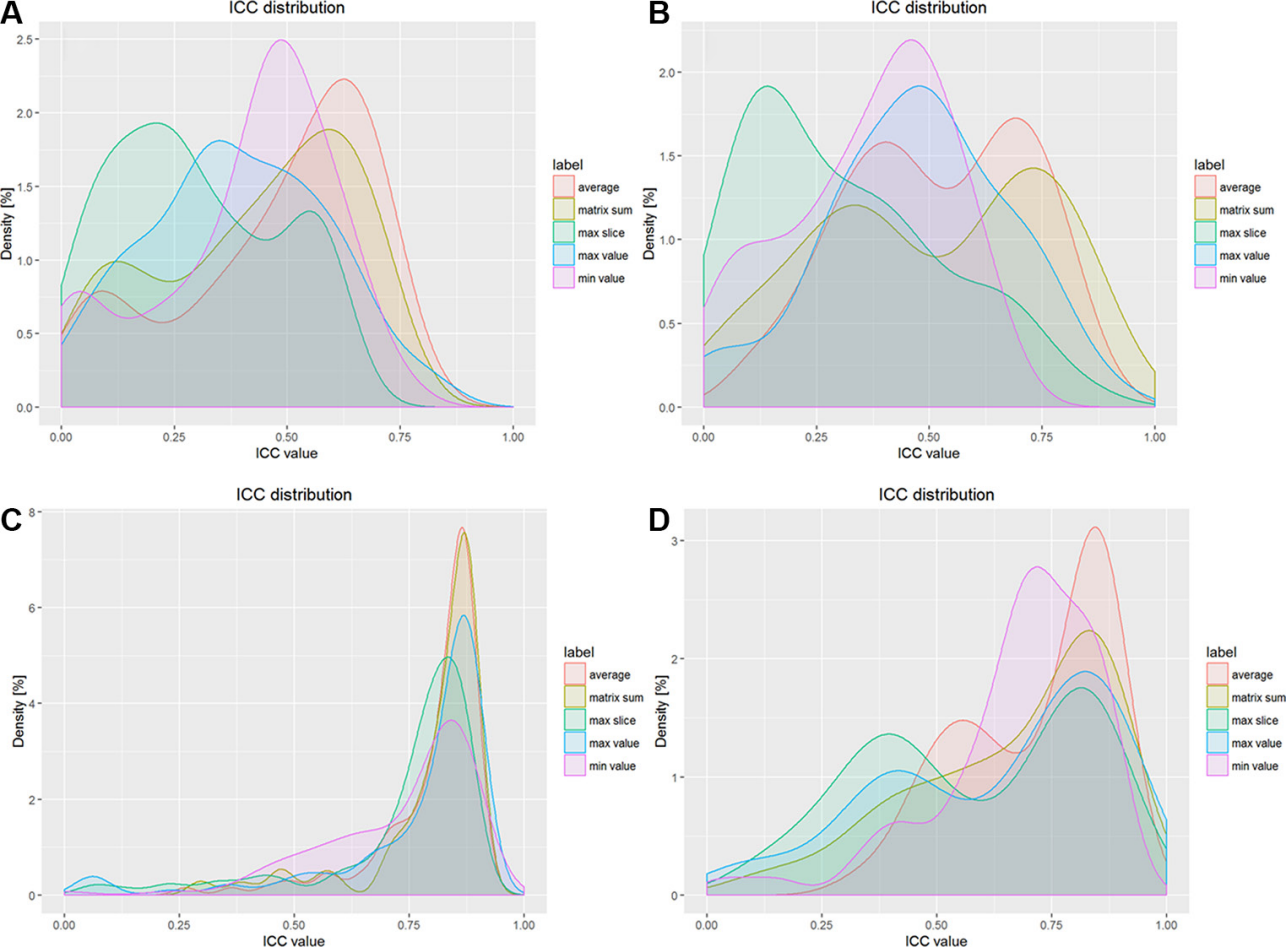
Results of ICC values for GLCM and GLRLM features (**A**) ICC values for unnormalized GLCM features, (**B**) ICC values for unnormalized GLRLM features, (**C**) ICC values for normalized CCC features, (**D**) ICC values for normalized ICC features.

In Figures 3 (A) and (B), the peak values of probability density for different types of original features are less than 2.5%, but in Figures 3 (C) and (D), the peak values of volume normalized features are 7.5% and 3 %, respectively. In Figure 3 (C), for GLCM features, average value type and matrix sum type are more stable than other types; for GLRLM features, average value type is the most reproduced of all.

**Figure 4:**
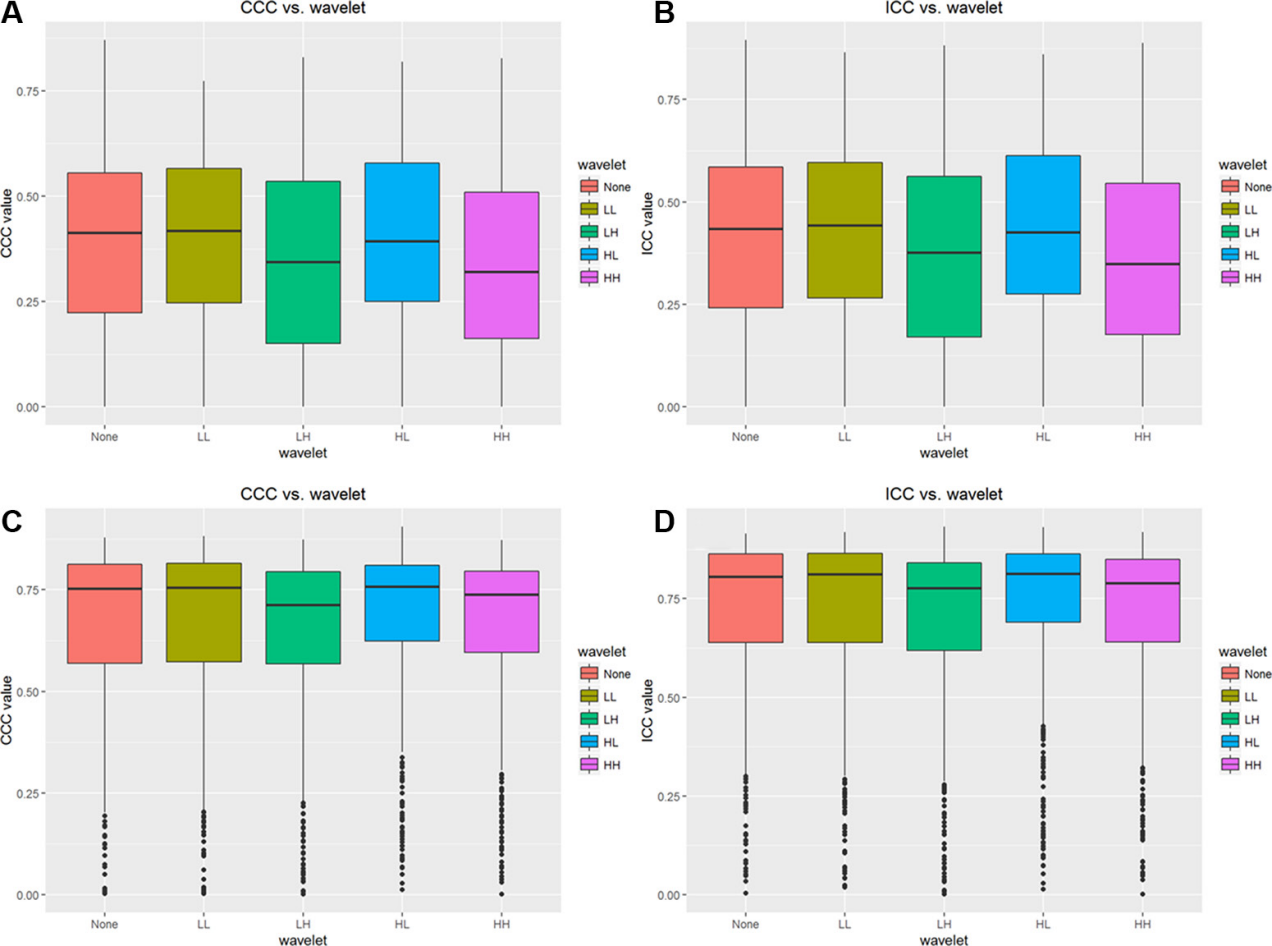
Results of CCC and ICC values for different wavelet features (**A**) CCC values for unnormalized wavelet features, (**B**) ICC values for unnormalized wavelet features, (**C**) CCC values for normalized wavelet features, (**D**) ICC values for normalized wavelet features.

**Figure 5:**
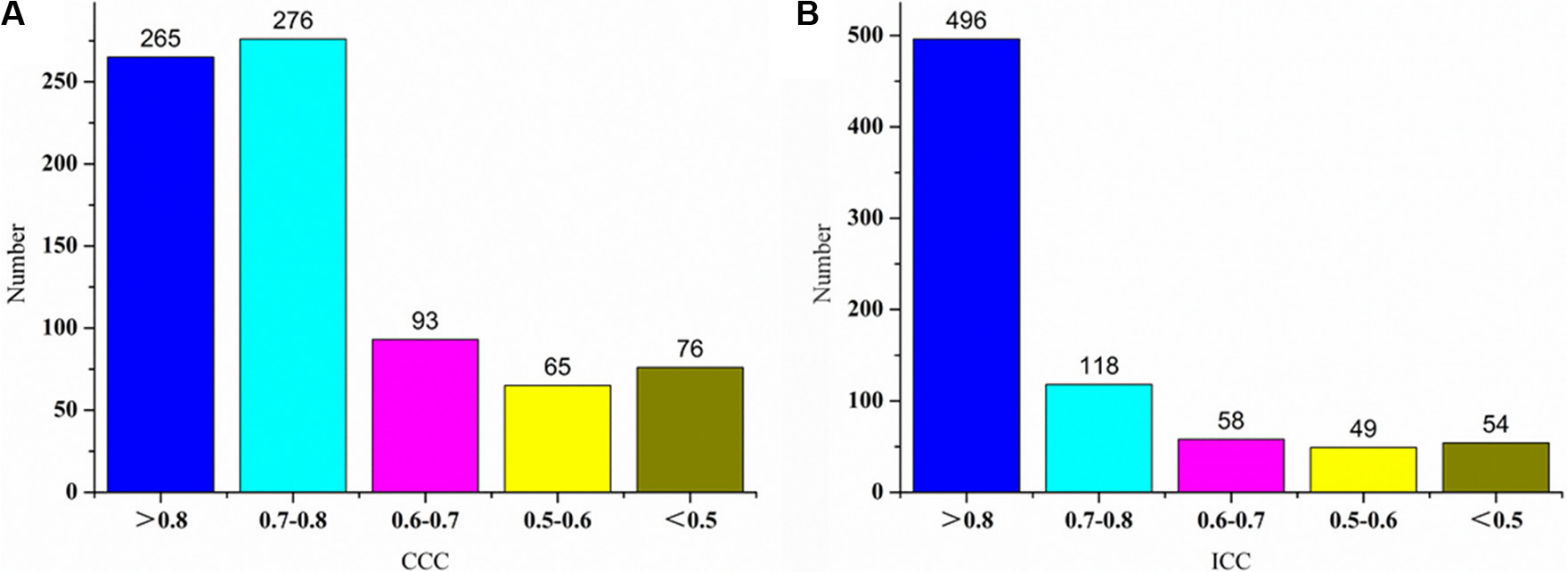
Histograms of CCC and ICC values of normalized features for the type of average value

Figure 4 illustrates CCC and ICC values with different wavelet characteristics for features, and the mean values for volume normalized features are much larger than unnormalized features. For volume normalized features, CCC and ICC values are all about 0.75 and 0.80, respectively. So there is no significant difference among wavelet features showed in Figure 5 (C) and (D).

Figure 5 shows histograms of CCC values and ICC values of volume normalized features for the type of average value. Most of features have high reproducibility. For the average type normalized features, 496 out of 775 features (64.0%) showed high reproducibility (ICC ≥ 0.8), 225 out of 775 features (29.0%) showed medium reproducibility (0.8 > ICC ≥ 0.5) and 54 out of 775 features (7.0%) showed low reproducibility (ICC < 0.5).

## DISCUSSION

Zhao et al. [7] analyzed variability from same-day repeat CT scans, quite a short wait time cannot analyze the reproducibility of radiomics, but this study evaluated the reproducibility of rectal tumor textural features from repeat CT scans which were underwent within average 8.7 days. Nowadays, radiomics studies for predicting prognosis are classified according to different stages of cancer, the interval from 5 days to 17 days showed in Table 2, and rectal cancer is still in the same stage. For a long interval, but textural features still have a good stability, the result has a higher reliability, and it indicates that CT image's textural features are highly stable and reproduced for rectal cancer.

**Table 2:** Characteristics of forty patients whose images were used in this study

Characteristics	Number of patients	Percent of patients (%)
Number of patients	40	NA
Median age (range)	50.5 (23–76)	NA
Median GTV volume (range) (cm^3^)	38.2 (2.3–171.3)	NA
Gender		
Male	24	60
Female	16	40
Days between two scans	8.7 (5–17)	NA

As we know, human's tumor is changing fast, different time the tumor may be different, to evaluate the reproducibility of radiomics study, the wait time of two CT scans should be a little longer. So an extended wait time is needed to analyze the reproducibility of radiomics features.

In this study, the values of the GLCM features were unnormalized and normalized to target volumes. The reason we use this normalization method is the connection between target volume and feature values. As slices area increase, more CT pixel will be included for analysis, and there will be more possibility to include some extreme value. But in GLCM matrix calculation, we use same bins (usually 32) to group these pixels. This will change the distribution shape of the pixel values. For example, in GLCM contract calculation, we use eq(1), where *P(i,j)* is the value of the pixel, *n* is the number of the pixels. As range of the CT value increase, the value of contract will decrease as more pixels will centralized.

$$f=\sum_{n=0}^{N_{g}-1}n^{2}\{\sum_{i}\sum_{\begin{array}{c} j\\ |i-j|=n \end{array}}P(i,j)\}$$ (1)

Furthermore, we analyzed the correlation between textural features from the type of average value and tumor volumes, ICC values between textural features and tumor volumes illustrate that 252 out of 775 features (32.5%) have a high correlation and 227 out of 775 features (29.3%) have a medium correlation. Besides, Zou et al. [8] also reported that textural features have a strong correlation with volumes. The results of this study indicated that volume normalized features are much more stable than unnormalized ones.

775 textural features were extracted from patients' medical images with rectal cancer, and we failed to find any statistically significant differences among each texture feature. It suggests that the further analysis of these textural features can be warranted for treatment monitoring and outcome prediction for rectal cancer. Furthermore, we innovatively compared the advantage and disadvantage of five types of patient's medical images. Additionally, the results indicated that different filters have little effect to textural features.

## MATERIALS AND METHODS

### Patients

Forty rectal cancer patients with stage II were included retrospectively in this study. All patients underwent two baseline clinical CT scans within average 8.7 days (5 days to 17 days) at Fudan University Shanghai Cancer Center in China, before any treatment was delivered. Both scans were obtained with the same CT scanner by using the same imaging protocol (350mA tube current, 120 kVp tube voltage, 0.92 0.92 mm pixel size, 5 mm section collimation, 512 512 matrix). These patients' medical images were divided into two groups: scans 1 and scans 2. The patients' characteristics are showed in Table 2.

### Contouring

The rectal GTV was distinguished and segmented by an experienced radiation oncologist in Eclipse (11.0, Varian, Palo Alto, CA), the delineation was double-checked and the non-invaded rectal wall and the air inside the rectum were carefully excluded. After contouring, the DICOM images and the DICOM contours were exported to MATLAB (Math works Inc, Natick, USA) for feature extracting and analysis.

**Figure 6:**
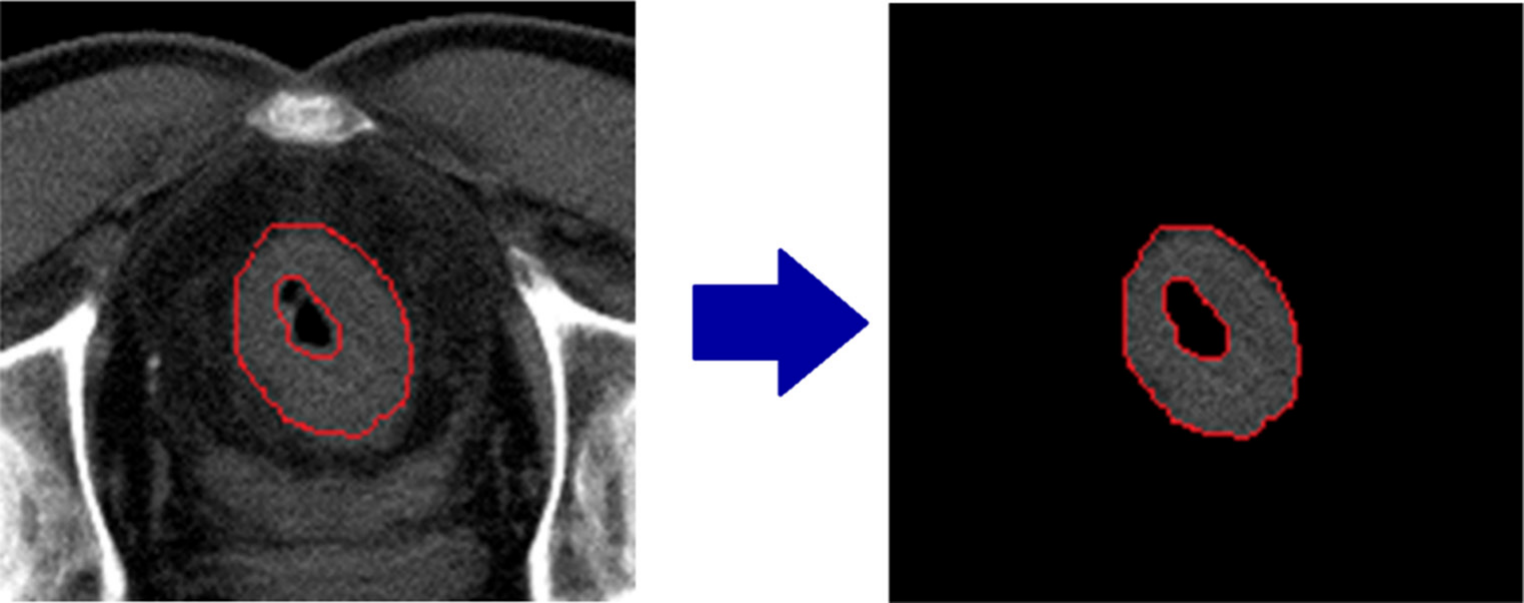
Contoured region cropping using binary mask

The contoured regions of the images were cropped from the whole patient CT image. This was realized by creating a binary mask base on contouring (Figure 6).

### Radiomics features extraction

We defined 775 radiomics image features to quantify tumor textural characteristics (detail of these features were list in Supplementary Table S1). These features were divided into four groups: I) GLCM [5] textural features, II) GLRLM [6] textural features, III) Wavelet GLCM textural features and IV) Wavelet GLRLM textural features. For each patient, all textural features were extracted from five types of patient's medical images, respectively: I) Max Slice, II) Max value, III) Min Value, IV) Average Value and V) Matrix Sum. All textural features were extracted via Matlab R2015a software (Mathworks Inc, Natick, USA). Zou's study shows lots of features were highly correlated with volume of the tumor [8]. So in this study original features and volume normalized features are both analyzed in this [3].

### Data analysis

To estimate the reproducibility and repeatability of the tumor textural features by using repeat CT data, the concordance correlation coefficient (CCC) and the intra-class correlation coefficient (ICC) were initially used [9, 10].

In statistics, the CCC measures the agreement between two variables, for example to evaluate reproducibility or for inter-rater reliability. McGraw and Wong [11] has the form of the concordance correlation coefficient as follow:

$$\text{CCC}=\frac{2\rho \sigma_{x}\sigma_{y}}{\sigma_{x}^{2}+\sigma_{y}^{2}+{\left(\mu_{x}-\mu_{y}\right)}^{2}}$$

Where μ_x_ and μ_y_ are the means for the two variables, σ_x_ and σ_y_ are the corresponding variances, ρ is the correlation coefficient between the two variables. R package IRR (inter rater reliability) was used for CCC computation [12].

Statistically, the ICC is a descriptive statistic that can be used when quantitative measurements are made on units that are organized into groups [13]. It ranges between 0 and 1, indicating null and perfect reproducibility. In order to determine the ICC for inter-observer segmentations, variance estimates were obtained from two-way mixed effect model of analysis of variance (ANOVA), Leijenaar [14] defined ICC as follow:

$$\text{ICC}=\frac{MS_{R}-MS_{W}}{MS_{R}+(k-1)MS_{W}}$$

Where MS_R_ = mean square for rows, MS_W_ = mean square for residual source of variance, k = number of observers involved and n = number of subjects. R package version 3.1.3 IRR was used for ICC computation [12].
